# Treatment Choices and Healthcare Services Utilization Amongst Lumbosacral Radiculopathy Patients: Results from a Randomized Controlled Trial

**DOI:** 10.21926/obm.icm.2503035

**Published:** 2025-08-07

**Authors:** Ryan S. Wexler, Natasha Parman, Devon J. Fox, Danielle ZuZero, Anand Parikshak, Sophia Kwin, Austin R. Thompson, Hans L. Carlson, Thomas Kern, Scott D. Mist, Ryan Bradley, Heather Zwickey, Courtney K. Pickworth

**Affiliations:** 1. Helfgott Research Institute, National University of Natural Medicine, Portland, OR, USA; 2. Chronic Pain and Fatigue Research Center, Department of Anesthesiology, University of Michigan Medical School, Ann Arbor, MI, USA; 3. Oregon Center for Complementary and Alternative Medicine in Neurological Disorders, Department of Neurology, Oregon Health & Science University, Portland, OR, USA; 4. School of Nursing, University of Washington, Seattle, WA, USA; 5. Department of Orthopaedics and Rehabilitation, Oregon Health & Science University, Portland, OR, USA; 6. Comprehensive Pain Center, Department of Anesthesiology & Perioperative Medicine, Oregon Health & Science University, Portland, OR, USA; 7. Herbert Wertheim School of Public Health and Human Longevity Sciences, University of California, San Diego, La Jolla, California, USA

**Keywords:** Back pain, radiculopathy, mindfulness, non-pharmacologic management

## Abstract

Lumbosacral radicular pain (LRP) is a common sequelae of low back pain, the world’s leading cause of years lived with disability. LRP typically causes numbness, weakness, and tingling into the lower extremity and is associated with high rates of pain and impaired function. Despite its prevalence, there is significant heterogeneity among clinical practice guidelines for the treatment of LRP, which may contribute to poor patient outcomes. The aim of the present study was to identify treatments that participants had previously attempted before enrolling in a randomized controlled trial of a mindfulness-based intervention. This analysis evaluated prior pharmaceutical use, procedures, and non-pharmacologic treatments, with a special focus on complementary and integrative health (CIH) utilization. The data for the present analysis were taken from the health history form of the baseline visit for the randomized controlled trial by Wexler et al 2024. Treatment utilization was evaluated and reported using descriptive characteristics. In this sample of chronic LRP patients (*n* = 71), we found a high proportion of CIH utilization, including acupuncture (58%), chiropractic care (58%), and herbs/supplements (42%). Most patients (52%) were utilizing two or more CIH modalities to manage their pain. A high percentage of participants had also previously used non-steroidal anti-inflammatories (61%) to manage their pain, and over a third of participants had previously undergone an epidural steroid injection (34%). In our trial, CIH utilization was much higher for treatments like chiropractic care, acupuncture, natural products, and physical activity than has been reported in previous large datasets of patients with chronic pain such as the National Health Interview Survey. Collecting data on CIH utilization in clinical trials can enable researchers to compare their samples to large national datasets and identify differences in use among specific populations. In addition, healthcare utilization data collected in clinical trials can further inform the development of clinical practice guidelines.

## 1. Introduction

Low back pain (LBP) is the leading cause of years lived with disability worldwide and has a significant negative impact on activities of daily living, absenteeism, and healthcare costs [1]. Many treatment options exist, including exercise, manual therapy, medications, spinal injections, and surgery [2]. Lumbosacral radicular pain (LRP), also known as spine-related leg pain [3], or colloquially as “sciatica”, is a common neuropathic comorbid condition associated with LBP characterized by numbness, tingling, and weakness into the lower extremity [3]. Lumbosacral radiculopathy is often considered a type of LRP caused by a lesion of the lumbar or sacral nerve roots, with neurologic sensory, motor, and/or reflex deficits in the corresponding nerve root distribution (dermatomes, myotomes) and occurs in roughly 3–5% of all patients, making it one of the most common reasons for patients to seek consults from neurologists and orthopedic surgeons [4, 5]. In about 25% of LRP patients, symptoms will persist for more than three months, moving this subset of patients into the category of chronic pain [6].

LRP typically occurs secondary to LBP; therefore, clinical practice guidelines of LBP sometimes address LRP treatment [7]. To collate these guidelines, previous teams have conducted systematic reviews for both pharmacologic and non-pharmacologic treatment of LRP and found that the methodologic quality of the evidence for LRP treatment is varied [8, 9]. There are conflicting recommendations amongst current clinical practice guidelines for non-invasive, pharmacologic, and invasive interventions such as physical activity, pain education, multidisciplinary treatment, and exercise/physical therapy [8, 9]. This inconsistency may lead to confusion or variability in treatment decision-making, both for providers and patients. As a result, many patients – particularly those with persistent symptoms – may seek care outside the conventional medical system, including complementary and integrative health (CIH) modalities. This behavioral pattern highlights the importance of characterizing CIH use in real-world clinical populations.

Despite the development of clinical practice guidelines, patients with chronic LRP, and chronic pain in general, may pursue alternative treatments due to factors including: patient and provider preferences, insurance coverage, knowledge about the guidelines, and availability of recommended treatments [10, 11]. Even with this higher patient utilization, complementary and integrative health (CIH) interventions have historically been under-evaluated in large data sets and practice-based research networks. The need for characterizing CIH practices and their application is critical when considering their increasing use in pain management [12]. Therefore, the objective of the present analysis is to characterize the conventional healthcare and CIH utilization of LRP patients who volunteered for a randomized controlled trial (RCT) of a mindfulness-based intervention.

This descriptive analysis offers value by characterizing how patients with chronic LRP navigate the healthcare system, often integrating CIH alongside conventional treatments. Understanding these patterns can inform the design of future trials, guide clinical decision-making, and help tailor interventions to the prior experiences and preferences of patients.

## 2. Methods

Data for this analysis originated from the RCT by Wexler et al. 2024 [13, 14]. The parent study evaluated Mindfulness-Oriented Recovery Enhancement (MORE) against treatment as usual (TAU) for patients with chronic LRP.

### 2.1 Recruitment

In the parent trial, adults with LRP were recruited from the National University of Natural Medicine Health Center, Oregon Health & Science University Spine Center, and Oregon Health & Science University Comprehensive Pain Center. Eligibility criteria relevant to this analysis were as follows: relevant ICD-10 code (Table 1), English-speaking, age 18–65, presence of radiculopathy symptoms extending below the knee for greater than six weeks at phone screening, no epidural steroid injection in the prior three months, no spine-related surgery in the prior six months, no concurrent diagnosis of cancer, no unmanaged or uncontrolled mental illness known to cause psychosis, and no regular mindfulness practice of at least once per week or any history of formal mindfulness training (e.g., previous mindfulness-based interventions or meditation retreats). Patients were initially contacted via email and followed up with via phone for telephone screenings. Study baseline visits were then scheduled to take place at Helfgott Research Institute in Portland, OR.

### 2.2 Data Collection

All data for the present analysis were collected in the parent trial’s health history questionnaire administered via REDCap at an in-person study visit. Questionnaires were delivered on a study iPad. The health history questionnaire was developed by the study team to be specific to LRP patients’ symptoms, current treatments, previous treatments, and CIH utilization.

### 2.3 Statistical Analysis Plan

Statistical analyses were conducted using SPSS Version 29.0.0.40. Data from the health history questionnaire are presented here using descriptive statistics. Categorical variables are presented as counts and proportions, and continuous variables are presented as means and standard deviations (SD).

## 3. Results

Data for the present analysis were collected in the parent study from January 2021 to January 2022. Seventy-one participants were enrolled at baseline and completed the health history questionnaire. This sample was predominantly white (81.7%) and female (64.8%), as is typical of CIH clinical trials [15–17]. All demographic data for this sample can be found in Table 2, and the proportion of participants having used previous treatments can be found in Table 3. To protect participant anonymity regarding treatment utilization, counts below three have been omitted.

The most reported conventional interventions previously used were physical therapy (73%) and NSAIDs (61%). Twenty-four (34%) of the subjects had undergone epidural steroid injections, while twelve (17%) had previously undergone surgery for LRP. In this sample of chronic LRP patients, we also found a high proportion of CIH utilization, including acupuncture (58%), chiropractic care (58%), and herbs/supplements (42%). Most patients in this sample (52%) currently used two or more CIH modalities to manage their pain, indicating a trend toward multimodal treatment strategies.

Due to the high degree of heterogeneity of treatments tried within this sample, we chose to combine some therapies into broader treatment categories (e.g., physical activity included yoga, stretching, strength/weight training, swimming, and cycling). Among participants who reported using physical activity to manage their pain, yoga was the most frequently reported form.

## 4. Discussion

This study examined the treatment history of adults with LRP who volunteered for a clinical trial involving a mindfulness-based intervention. Our findings show that our sample had a high degree of CIH utilization despite many of these interventions having conflicting recommendations in published guidelines. We hypothesize that this is due to the long condition duration in this sample, over 13 years [14], which may have driven many patients to seek alternative forms of pain management. Although the success of prior treatments was not directly assessed, the long condition duration in our sample and extensive history of prior treatments suggest participants may have sought mindfulness as a later-stage or alternative strategy after previous efforts provided insufficient relief. Compared to the National Health Interview Survey, a large, nationally representative survey conducted by the U.S. Census Bureau, our sample reported higher use of chiropractic care, acupuncture, natural products, and physical activity [18]. This difference may have been due to a volunteer bias in that participants interested in a trial of a mindfulness-based intervention may be more likely to utilize CIH treatments already.

The demographic characteristics of our sample – predominantly female and white – align with previously reported trends in CIH use. Prior national surveys have shown higher rates of CIH engagement among women and white adults [16, 17]. These demographic patterns may reflect broader sociocultural, economic, and healthcare access factors that influence care-seeking behavior and preference for non-pharmacologic treatments. However, they may also limit the generalizability of our findings to more diverse patient populations. Previous systematic reviews of clinical practice guidelines have exemplified the array of treatment recommendations that exist for LRP. Khorami et al. published a systematic review examining 23 international guidelines for the management of LRP. Taking the guidelines’ recommendations together, they concluded the following treatments have conflicting recommendations: bed rest, acupuncture, traction, manipulation/mobilization/soft-tissue techniques, massage, ultrasound, heat/cold/infrared therapies, medications (including paracetamol, NSAIDs, opioids, anticonvulsants, muscle relaxants, antidepressants, corticosteroids, and antibiotics), and epidural injections [9]. Despite these conflicting recommendations, over half of our sample reported using acupuncture, NSAIDs, and/or chiropractic care (i.e., mobilization/manipulation). Another review by Price et al. summarized guidelines regarding pharmacological interventions specifically [8]. Eleven clinical practice guidelines were included, and the authors reported little agreement among the guidelines. They also found that three guidelines specifically recommended against pharmacologic interventions due to a lack of supporting literature [8]. These findings add to the literature base indicating that patients often use interventions not supported by high-level evidence such as systematic reviews.

While many treatments for LRP carry conflicting recommendations across guidelines, there is a consensus supporting non-invasive, non-pharmacologic strategies (e.g., physical activity, patient education, and multidisciplinary rehabilitation) as first-line care. Mindfulness-based interventions are increasingly recognized as part of a biopsychosocial approach to chronic pain care, though their formal inclusion in clinical guidelines is still emerging [19]. The growing presence in research-supported care models reflects an evolving understanding of the role of psychological and behavioral therapies in managing persistent pain conditions like LRP.

In attempting to compare our study cohort to other non-review studies, we found some studies did not aim to characterize prior treatments or used different clinical populations. For example, in the retrospective analysis conducted by Trager et al., the aim was to determine the association between spinal manipulation and lumbar spine surgery. In a separate analysis, the same team examined the likelihood of tramadol prescription in those who received spinal manipulation compared to those who did not [20, 21]; however, other CIH utilization was not evaluated. Golubovsky et al. compared the treatment history of those with Bertolotti syndrome to those with LRP in a much smaller cohort, but only inquired about epidural injections and previous surgery and not about other types of conventional biomedical interventions or CIH [22]. Thus, a strength of the current analysis is the breadth of described interventions and treatment modalities compared with other studies.

To compare our study against other RCTs, we were able to identify two RCTs presenting treatment history data for LRP patients. One RCT examined previous medication use in a sample of 60 patients attending a university hospital pain management center. The trial intervention was epidural steroid injections. They found 40.0% of their sample had previously used NSAIDs, 23.3% had previously used anticonvulsants, and 15% had previously used antidepressants for their lumbosacral radiculopathy symptoms [23]. However, other interventions were not reported. Our sample had a greater percentage who had used NSAIDs (61% compared to 40%), and smaller percentages who used anticonvulsants and SNRIs. The second trial, also conducted at an interventional pain management center, evaluated 56 patients with lumbosacral radiculopathy due to epidural fibrosis and found that 35.7% had previously used NSAIDs. Again, no other treatment history beyond medication use was reported [24], and our cohort had a greater percentage who used non-steroidal anti-inflammatories.

Lastly, we identified one Delphi study which aimed to develop consensus regarding conservative treatment based on LRP stage (i.e., acute, sub-acute, chronic). The study recommended several treatments for chronic LRP: spinal manipulation, specific exercises, and function-specific exercises should be combined with individualized vocational, ergonomic, and postural advice [25]. They also recommended patient education, physical activity, pain medication, and injections be utilized in earlier stages of LRP. Additional CIH interventions were not listed in their recommendations.

It is worth noting that comparing our findings to other studies is difficult for numerous reasons. First, our study specifically recruited patients with LRP. Depending on the study aim, the presence of radicular symptoms is sometimes an exclusion criterion in studies of patients with LBP. In addition, while surgery is sometimes an exclusion criteria in other studies of LRP or LBP, we allowed patients who had previously had lumbar surgery greater than 6 months prior to enrollment with ongoing symptoms [26]. Lastly, many studies do not report their subjects’ treatment history, or the history is focused on one type of intervention and not all interventions tried, whereas we aimed to be comprehensive in our collection and reporting of previous treatments and CIH utilization.

### 4.1 Limitations

Our study has a number of important limitations relative to our aim. While we tried to be comprehensive when designing the health history form, we did not inquire about specific pain medications apart from NSAIDs or opioid prescriptions. We also do not have details on when previous treatments were tried relative to patients’ study enrollment or if treatments were delivered in an integrative manner (i.e., in combination). We do not have detailed information on treatment dose (e.g., dose of herbal supplements, number physical therapy sessions). Treatment history was self-reported and not confirmed via medical record reviews. This was a relatively small sample of patients (*n* = 71) and patients were geographically located in the Pacific Northwest region. In addition, part of this sample (*n* = 11) was recruited from a CIH academic medical center; thus, these patients may have been more likely to engage in CIH practices. Participants with regular mindfulness practice or prior formal mindfulness training were excluded from the trial (*n* = 25 during recruitment screening). This exclusion criterion may have biased the sample toward individuals less familiar with mindfulness-based CIH interventions, thereby limiting generalizability to populations with prior mindfulness exposure. Finally, any self-report measure is affected by errors of recall. This is especially relevant in samples of patients with chronic pain as these patients have frequently attempted a variety of treatments over the course of multiple years (e.g., chiropractic treatment concurrently with physical therapy, multiple attempts at physical therapy, multiple attempts at acupuncture).

### 4.2 Future Directions

Large longitudinal cohort studies and practice-based research networks have the ability to capture important data about CIH utilization amongst patients with chronic pain. Currently, this information is captured in large datasets such as the National Health Interview Survey but is not routinely collected in clinical trials. In future chronic pain clinical trials, we aim to continue refining the capture of past and current CIH utilization as a model for how this might be implemented in larger healthcare settings. One recent example of this comes from the Pain Management Collaboratory, an inter-agency initiative between the National Institutes of Health, Department of Defense, and Veterans Affairs. This group identified a lack of standardized questionnaire for the capture of pain-self management strategies and developed the Nonpharmacological and Self-Care Approaches Measure from the Pain Management Collaboratory (NSCAP) tool to collect this information. This tool may be used in future clinical trials to standardize the collection of pain treatment information and is already being used in trials with the Pain Management Collaboratory [27–29]. In addition to actual treatment history, future intervention studies should consider the capture of participants’ perceived effectiveness and treatment expectancy for CIH interventions as these datasets can meaningfully contribute to systematic reviews and the development of clinical practice guidelines.

## 5. Conclusion

In our trial of patients with lumbosacral radicular pain, CIH utilization was much higher for treatments like chiropractic care, acupuncture, natural products, and physical activity than in previous large datasets of patients with chronic pain [18]. Collecting data on CIH utilization in clinical trials can enable researchers to compare their samples to large national datasets and identify differences in use among specific populations, providing valuable insights that will drive updated evidence-based clinical practice guidelines for CIH and non-CIH providers alike.

## Figures and Tables

**Table 1:** ICD-10 Code Descriptions.

M54.16	Radiculopathy, lumbar region
M54.17	Radiculopathy, lumbosacral region
M51.16	Intervertebral disc disorders with radiculopathy, lumbar region
M51.17	Intervertebral disc disorders with radiculopathy, lumbosacral region
M47.26	Other spondylosis with radiculopathy, lumbar region
M47.27	Other spondylosis with radiculopathy, lumbosacral region
M54.40	Lumbago with sciatica, unspecified side
M54.41	Lumbago with sciatica, right side
M54.42	Lumbago with sciatica, left side
M99.53	Intervertebral disc stenosis of neural canal of lumbar region
M99.54	Intervertebral disc stenosis of neural canal of sacral region
S34.21	Injury of nerve root of lumbar spine
S34.22	Injury of nerve root of sacral spine
G54.4	Lumbosacral root disorders, not elsewhere classified
G55	Lumbosacral root disorders, not elsewhere classified

**Table 2:** Demographic characteristics of 71 participants at baseline presented as mean (±SD) or n (%).

Age	46.85 (±11.59)
Race	
White	*n* = 58 (82)
Black	*n* = 2 (3)
Asian	*n* = 2 (3)
Middle Eastern	*n* = 2 (3)
More than one race	*n* = 5 (7)
Other/unknown/missing	*n* = 2 (3)
Sex	
Female	*n* = 46 (65)
Male	*n* = 25 (35)

**Table 3:** Frequency of Treatment Utilization Amongst 71 Participants in the Parent RCT.

**Procedures**	
Epidural Steroid Injection	*n* = 24 (34)
Surgery	*n* = 12 (17)
Nerve Blocks	*n* = 7 (10)
**Pharmaceuticals**	
Non-steroidal anti-inflammatories (NSAIDs)	*n* = 43 (61)
Opioids	*n* = 9 (13)
Steroids	*n* = 7 (10)
Muscle Relaxers	*n* = 4 (6)
Anticonvulsants	*n* = 4 (6)
**Natural Products**	
Single Herbs/Supplements	*n* = 27 (38)
CBD and Cannabis	*n* = 7 (10)
Combination Supplements	*n* = 4 (6)
Homeopathic Remedies	*n* = 4 (6)
**Manual Modalities**	
Physical Therapy	*n* = 52 (73)
Acupuncture/Acupressure	*n* = 42 (59)
Chiropractic Care	*n* = 41 (58)
Craniosacral Therapy	*n* = 8 (11)
Massage	*n* = 5 (7)
TENS	*n* = 4 (6)
**Other Treatments**	
Physical Activity	11 (15)
Other	5 (7)

To protect participant anonymity regarding treatment utilization, counts below three have been omitted.

## Data Availability

The data that support the findings of this study are available from the corresponding author, RSW, upon reasonable request.
